# Effect of pH on temperature controlled degradation of reactive oxygen species, heat shock protein expression, and mucosal immunity in the sea cucumber *Isostichopus badionotus*

**DOI:** 10.1371/journal.pone.0175812

**Published:** 2017-04-17

**Authors:** Mariel Gullian Klanian, Montserrat Terrats Preciat

**Affiliations:** Experimental Unit, Marist University of Mérida, Mérida, Yucatán, México; University of Catania, ITALY

## Abstract

This study evaluated the effect of pH on the activity of antioxidant and immune enzymes in the sea cucumber *Isostichopus badionotus* exposed to different temperatures. The organisms (530 ±110 g) were exposed to 16, 20, 24, 28, 30, 34 and 36°C for 6 h to evaluate thermal limits at two water pH values (treatment = 7.70; control = 8.17). For the thermal tolerance experiment, the organisms were exposed to sublethal temperature of 34°C for 3, 6, 12, 24, and 48 h. *I*. *badionotus* showed signs of thermal stress by synthesizing heat shock protein 70 (hsp70) at the cold (16°C) and warm thermal limits (34°C). The glutathione peroxidase (GPx) showed a negative correlation with superoxide dismutase (SOD) activity in modulating the effect of oxidative stress at different temperature levels. Specifically, GPx activity was maximal at the extremes of the cold and warm temperatures (16, 20, and 36°C) tested, while contrarily, the SOD activity increased significantly in the narrow range of temperature between 28 and 30°C, as a part of a reaction to offset oxidative damage. The effect of pH on the expression of hsp70 was not significant, whereas the antioxidant enzymes activity was stimulated at pH 7.70. Mucosal immunity, evidenced by the activation of the phenoloxidase (PO) system, increased above the basal level at pH 7.70 and at 28, 30, and 34°C. Independent of pH, the temperature of 34°C was identified as the 12 h-sublethal upper limit for *I*. *badionotus*.

## Introduction

The sea cucumber *Isostichopus badionotus* (Echinodermata: Holothuroidea) is a large epibenthic holothuroid distributed along the Western Atlantic coast from South Carolina to Brazil, including the Antilles, and the Gulf of Guinea in the Eastern Atlantic [1, 2]. In the last ten years, *I*. *badionotus* has been subject to intense fishing in the southern Gulf of Mexico, the Caribbean islands, and the Central American countries due to a high demand in Asian countries. Since then, many efforts have been made to stabilize its aquaculture such as contributing knowledge of its vulnerability to different environmental conditions. In their natural environment, sea cucumbers from southern Gulf of Mexico are exposed to seasonality, which often results in fluctuations in water pH that ranges between 7.3 and 8.6 [3, 4]. The dry season is characterized by a high water temperature and low water movement that result in low pH values between 7.3 and 7.5, while the pH increases to 8.4–8.6 during the rainy season due to a high influx of freshwater effluent. During the north wind season (October to January), the water temperature decreases from a maximum of 32°C to an average of 21°C [5].

The environmental factors play an important role in the physiology of sea cucumbers as they are sensitive to changes in temperature and water pH. Specifically, the functional relationship between antioxidant enzymes activity and cellular redox homeostasis is believed to be a critical regulator of thermal stress. In contrast to the sea cucumber from temperate climates *Apostichopus japonicus*, limited scientific information is available on *I*. *badionotus* regarding its vulnerability to temperature and pH fluctuations. Previous studies have demonstrated that decreasing water pH (5 to 10) and temperature affects the activity of immunological enzymes and phagocytosis rate in sea cucumbers. The phagocytic index and lysozyme activity increase in the intestine of juvenile *A*. *japonicus* exposed to alkaline pH while acid phosphatase activity decreases in the body wall [6]. The catalase activity (CAT) decreased significantly at 24°C after 0.5 h exposure and increased significantly at 32°C after 3 to 12 h; superoxide dismutase activity (SOD) also decreased after 1 h of thermal exposure but increased after 12 h [7]. As the metabolic rate increases, an estimated 2–3% of the oxygen consumed by aerobic cells is converted to oxygen free radicals (O_2_^−^) and hydrogen peroxide (H_2_O_2_), and greater tissue oxygen consumption presumably, leads to elevated rates of reactive oxygen species (ROS) production in the mitochondria [8,9]. The capacity of oxygen delivery completely matches to the aerobic scope only within the thermal optimum. At temperatures outside this range, only time-limited survival is supported by a residual aerobic scope. The subsequent effects include the induction of anaerobic metabolism and molecular protection by the heat shock proteins (hsp) and antioxidant defenses [10]. Generally, hsp expression can be correlated with the levels of stress experienced in nature; thus, for the sea cucumber, hsp70 is considered a specific bioindicator of thermal stress [11]. Thermal stress (30°C for 2 h) induces the expression of hsp70 in *A*. *japonicus* that reaches a maximum after 6 h of exposure [12].

Although pH is an important factor that affects the immunity of invertebrates, its effect on the antioxidant system and the expression of hsp70 in the sea cucumber have been poorly explored. In this context, the present study aimed to evaluate the effect of pH on the synthesis of hsp70, as well as the role of this environmental variable in stimulating immune response and antioxidant enzymes activity in the sea cucumber *I*. *badionotus* exposed to different temperature. The study also provided information on thermal limits and the thermotolerance of *I*. *badionotus* exposed to sublethal temperatures.

## Materials and methods

### Animal collection and acclimation

The organisms used in this study were lawfully obtained with the fishing permit PPF/DGOPA–026/13 (CONAPESCA, México). A total of 150 sea cucumbers (*I*. *badionotus*) weighing 530 ±110 g were randomly collected from the Telchac Puerto, Yucatán, Mexico (21° 21′ N, 89° 13′ W) by scuba diving to a depth of 10 to 28 m in October 2013. Sediment was extracted from the sample site using a box sediment sampler (Kahlsico 214WA170). The organisms were immediately transferred to the CINVESTAV marine station in Telchac (Yucatán, México), where they were kept under indoor laboratory conditions. At the laboratory, the sea cucumbers were divided into three batches and maintained for 6 days in 0.83 m^3^ indoor fiber glass tanks containing 5 cm of sediment from the collection site. The seawater, before being pumped into the tanks, was filtered, UV-sterilized, and adjusted to 26°C in a controlled environment. For the experiment, water parameters in the control treatment tank were maintained at those found at the collection site, i.e., 26°C, pH 8.17, and 35 psu with a light-dark cycle of 14 h and 10 h, respectively. The organisms were fed daily on a natural diet obtained from the collection site sediment.

### Experimental design

Two experiments were performed to evaluate the thermal tolerance limits (experiment a) and the thermal tolerance time (experiment b) at different pH values (experiment b). Prior to the exposure to different temperatures, 50% of the organisms were transferred to another tank and gradually exposed to pH(*t*) 7.70 and 26°C for 8 days. The remaining organisms were maintained at pH(*c*) 8.17 and 26°C (pH and temperature of the collection site).

For assessing thermal limits (experiment a), 40 individuals from each pH acclimation tank were randomly placed into 20 L experimental units (n = 5) and exposed to 16, 20, 24, 28, 30, 34 and 36°C for 6 h. The organisms from the control group were placed in the two experimental units (n = 5 for each time point) and maintained at pH 8.17 and 26°C for 6 h. For the thermal tolerance experiment (experiment b), 30 organisms from each pH were exposed to a sublethal temperature (34°C) for 3, 6, 12, 24 and 48 h (n = 6, for each time point). The thermal limit was calculated as control temperature ± 10°C in order to obtain a range of thermal exposures. The sublethal temperature was selected on the basis of the results of the thermal limit experiment.

After exposure, coelomic liquid (CL) and tissue samples were obtained and the organisms were re-acclimatized to control conditions to evaluate mortality over a period of 3 days.

### Monitoring and control of water parameters

The water pH in the treatments (pH(*t*) = 7.70 ±0.08) was manipulated by the addition of NIST-traceable CO_2_ (INFRA, Mexico). CO_2_ partial pressures (*p*CO_2_) of 1200–1400 μatm equivalents at pH 7.70 were maintained using a feedback system, which regulates pH by the addition of gaseous CO_2_. As the water flowed into the thermal treatments, CO_2_ was added back to reach a set point that was continuously monitored *in situ* with a probe equipped with a pH/mv/°C electrode (accuracy ±0.01 pH units and ±0.2°C; Oakton Instruments pH/CON 510, Vernon Hills, IL). Each experimental unit was independently handled to completely control pH and temperature parameters. The target temperatures above the ambient conditions were achieved using thermostatic immersion heaters in the experimental chambers, while the temperatures below the ambient conditions were maintained by placing the experimental units in an insulated chamber containing an ice-water recirculating bath. After exposure to the target temperatures, the sea cucumbers were returned to 26°C ± 0.2°C for 3 day to record mortality. During the experiments, the temperature was monitored at 20-min intervals using a waterproof digital thermometer (ETI 810–920, Worthing, UK). Aeration was continuously provided to maintain the amount of dissolved oxygen in the tanks at 6.57 ± 0.3 mg L^–1^ (YSI 550A, OH, USA). The mean of pH and temperature registered during the experimental period are shown in Table 1. The values of *p*CO_2_ (μatm) were estimated for each temperature according to the equation of Zeebe & Wolf-Gladrow [13], taking into consideration the following seawater quality parameters: dissolved inorganic carbon (DIC) = 2.1 mmol kg^–1^ and Ca^2+^ = 10 mmol kg^–1^.

**Table 1:** Mean of pH and temperature of the experimental units registered during 6 h of experimental time.

Temperature treatments (°C)		pH treatments			
Mean	SD	pH*(c)*	*p*CO_2_ (μatm)*	pH*(t)*	*p*CO_2_ (μatm)*
16	0.23	8.17	302	7.78	787
20	0.22	8.16	313	7.75	862
24	0.21	8.13	338	7.75	873
28	0.24	8.19	287	7.62	1223
30	0.25	8.18	292	7.61	1235
34	0.28	8.16	303	7.61	1242
36	0.20	8.16	295	7.60	1260
(Control) 26	0.24	8.19	356	-	-
		8.17 ± 0.02		7.70 ± 0.08	

pH*(c)* = pH of control; pH*(t)* = pH modified

Values of pH*(c)* and pH*(t)* columns are means of experimental data; n = 36.

*Values of *p*CO_2_ (μatm) were estimated for each temperature according to the equation of Zeebe & Wolf-Gladrow [14].

### Sample collection

After the exposure, the organisms were anesthetized using 0.5% magnesium sulfate [14]. CL (2 mL per organism) was withdrawn using a 3 mL syringe from the body wall of the organisms. Outer epidermal (100 mg) and muscle (400 mg) samples were also obtained through a non-lethal tissue biopsy. CL and tissue were thoroughly frozen in liquid nitrogen and stored at −80°C for further analyses. CL was used to measure total SOD and glutathione peroxidase (GPx) activity while the tissue samples were used to measure CAT, phenoloxidase (PO), and lysozyme (LSZ) activity and quantify hsp70 expression.

### Hsp70 extraction and quantification

The sample of muscle tissue was homogenized in 1.5 mL phosphate buffered saline (PBS). The homogenates were precipitated with 10% trichloroacetic acid and centrifuged at 2500 ×*g* for 10 min at 4°C. The supernatants were discarded and the pellets were re-homogenized for 15 s in 1.5 mL PBS. Hsp70 was quantified using an enzyme-linked immunosorbent assay (ELISA) in 96-well polystyrene plates (F96 Polysorp, Nunc), as follows [15]. The wells were coated with 50 μL of the sample (2.5 μg protein) for 2 h at 37°C and incubated at 4°C overnight. After two washes with PBS-Tween 20 (10 mM PBS, 0.05% Tween 20), the wells were blocked for 2 h at 37°C with blocking buffer (5% non-fat milk in PBS). The plates were washed twice with PBS-Tween 20 and 100 μL of anti-hsp70 primary antibody (1:2000 in PBS; Sigma-Aldrich, MO, USA) was added. The antibody localizes both the constitutive (HSP73) and inducible (HSP72) forms of HSP70. After incubation at 37°C for 2 h, the plates were washed thrice with PBS-Tween 20, 100 μL of peroxidase-conjugated goat anti-mouse antibody (Sigma-Aldrich, MO, USA) was added to the wells, and the peroxidase reaction carried out in 100 μL of phosphate-citrate buffer (0.1 M) containing 0.05% ABTS (2.2-azino-bis 3-ethylbenzo-thiazoline–6-sulphonic acid) and 0.1% H_2_O_2_. After 120 min of incubation at 37°C, the optical density was determined at 405 nm. For quantification, an eight-point calibration standard curve (0–10 ng mL^–1^ protein) was constructed using hsp70 active protein (Acris Antibodies GmbH, Herford, Germany).

### Antioxidant enzymes

The samples of frozen CL were thawed at 4°C, centrifuged at 3000 ×*g* and 4°C for 10 min to separate the supernatant from the packed cells, and the supernatant used to measure total soluble protein and antioxidant enzymes. The total protein from the CL and tissue samples was determined by the Lowry method for normalization [16].

SOD activity was measured as its ability to inhibit the formation of superoxide anions generated by the xanthine and xanthine oxidase (XO) reaction system [17]. The assay was performed with 50 μL of CL diluted to 200 μL Dojindo’s water-soluble tetrazolium salt (Sigma-Aldrich, MO, USA) and 50 μL of enzyme working solution (74043, Sigma-Aldrich, MO, USA). One unit of SOD was defined as the amount required for 50% inhibition of the rate of xanthine reduction in a coupled xanthine and XO system at 37°C [18]. SOD activity was measured at 450 nm in a microplate reader using a kinetic mode (Thermo Scientific, Multiskan EX, Rockford, IL, USA) and activity was expressed as units of SOD per mg of protein.

GPx activity was measured by the method of Mannervik [19]. The reaction was performed at 25°C by adding 300 μM of tert-butyl hydroperoxide (t-Bu-OOH) solution to 50 μL of CL diluted in 850 μL of GPx buffer (50 mM Tris-HCl, 0.5 mM EDTA) along with 50 μL of 5 mM β-nicotinamide adenine dinucleotide phosphate (NADPH) reagent, 42 mM GSH, and 10 U mL^–1^ of glutathione reductase. Activity was measured at 340 nm in a microplate reader using the kinetic mode (Thermo Scientific, Multiskan EX, Rockford, IL) and one unit of GPx was defined as the amount of enzyme required to oxidize 1 nmol NADPH to NADP^+^ per minute; GPx activity was expressed as units of GPx per mg of protein.

CAT activity was measured based on the rate of decomposition of H_2_O_2_, which is proportional to the reduction of absorbance at 240 nm [20]. Briefly, a tissue sample (5 g) was homogenized in 0.05 M PBS (pH 7.0), the homogenate centrifuged at 1,000 ×*g* for 10 min, and 2 mL of supernatant (2 mL) mixed with 1 mL of 30 mM H_2_O_2_ in PBS. The rate of decomposition of H_2_O_2_ was monitored every 30 s for 2 min in a spectrophotometer (Genesys 10S UV-Vis, Thermo Scientific, WI, USA). One unit of CAT was defined as the amount necessary to decompose 1 μM of H_2_O_2_ to oxygen in 1 min at 22°C. CAT activity was expressed as units of CAT per mg of protein.

### Immune enzymes

The tissue samples containing mucus and the epidermal layer of *I*. *badionotus* were removed from −80°C storage and macerated in liquid nitrogen. One part of the ground powder was mixed with three parts (w/v) of 0.1 M sodium phosphate buffer containing 1 M NaCl, 0.2% Brij 35 and 2% polyvinylpolypyrrolidone (PVPP). The suspension was stirred for 3 h at 4°C and centrifuged at 2000 ×*g* for 30 min, and the supernatant was used for PO and LSZ activity determination.

PO activity was assessed according to the method described by Söderhäll et al. [21] with recommendations from Jiang et al. [22]. Prophenoloxidase (PPO) was extracted according to the method of Simpson et al. [23], but with slight modifications. The reaction mixture containing 50 μL of PPO extract was incubated with 50 μL of 0.01 M sodium cacodylate buffer containing lipopolysaccharide (LPS, Sigma, MO, USA) as an inductor in 0.01 M calcium chloride and 0.26 M magnesium chloride (pH 7.0) in microplate wells and incubated at 26°C for 30 min. Next, 50 μL of 15 mM DL-3,4-dihydroxyphenylalanine (DL-DOPA) in 0.05 M Tris-HCl buffer was added to each well and absorbance was measured spectrophotometrically at 475 nm every 30 s for 10 min. One unit of PO activity was defined as the amount of enzyme that leads to a 0.001 increase in absorbance per min.

LSZ activity was quantified by measuring the ability of the sample to degrade a suspension of the bacterium *Micrococcus lysodeikticus* (M0508; Sigma, MO, USA) according to a modified method described by Lee & Yang [24]. The bacteria were resuspended in potassium phosphate buffer (0.1 M, 0.09% NaCl), and the optical density was adjusted to 0.7 absorbance units at 570 nm. The sample (50 μL) was placed in a 96-well plate at 37°C along with 150 μL of the *M*. *lysodeikticus* cell suspension. After shaking the plate, the reduction in turbidity was spectrophotometrically recorded for 4 min at 570 nm. The LSZ activity unit (U) was defined as the amount of enzyme required to produce a 0.001 min^–1^ change in turbidity under the conditions described above. LSZ activity was expressed in units and adjusted for protein concentration.

### Data analysis

The data (S1 and S2 Tables) were analyzed using the SPSS statistical package for Mac (SPSS, Chicago, IL, USA). Significant differences in biochemical variables among acclimation temperatures and pH were determined using the two-way ANOVA with interactions (Model I) followed by Tukey’s HSD multiple comparison test. The differences were considered significant at *P* < 0.05.

## Results

### Thermal thresholds

At 6 h of exposure and temperatures from 16 to 34°C, there were no mortalities in *I*. *badionotus*. However, 100% mortality rate after 3 h of exposure was observed in the organisms exposed to 36°C at both pH treatments. Thus, 34 and 36°C were defined as sublethal and lethal temperature, respectively. At sublethal temperature, 100% survival was found after 3, 6, or 12 h of exposure and it reduced to 83% after 24 h and 50% after 48 h.

### Expression of hsp70

**Fig 1:**
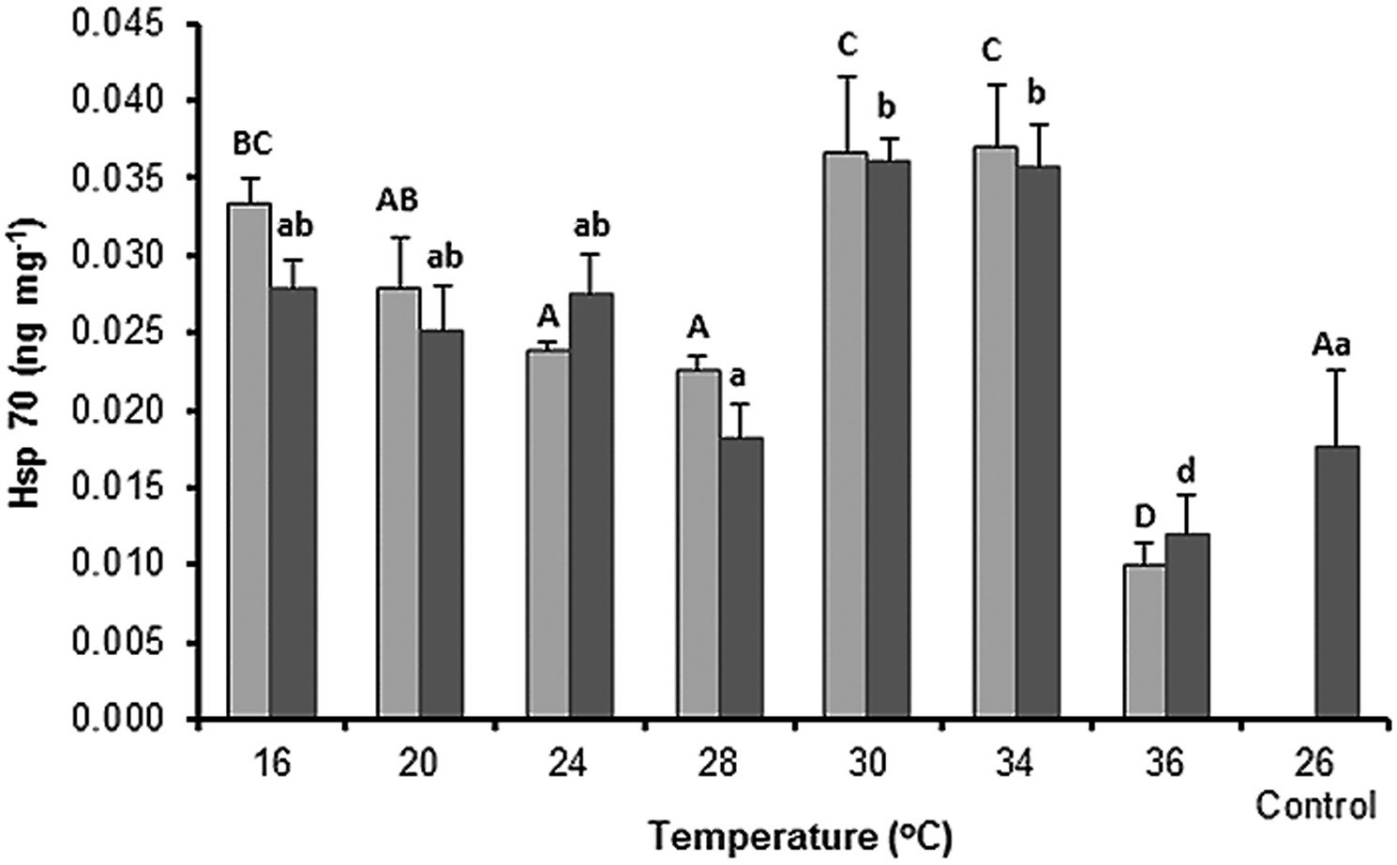
Effect of temperature on the expression of heat shock protein 70 (hsp70) from the sea cucumber *Isostichopus badionotus* exposed to two pH values. ■ pH 8.17 ±0.02; ■ pH 7.70 ±0.08; control conditions: pH 8.17 ±0.02, 26 ±0.2°C. Columns represent mean ±SE; n = 5 with the exception of the control where n = 10 (factorial ANOVA; Tukey’s HSD, *P* < 0.05). Different letters (lower case for pH 8.17; capital letters for pH 7.70) represent significant differences between temperatures.

Alterations in pH had no significant effects on hsp70 expression in organisms exposed to the same temperature (*F*_1, 64_ = 0.85, *P* = 0.290; Fig 1). The organisms from both pH treatments showed signs of thermal stress at 30 and 34°C, as hsp70 expression was significantly higher than control (*F*_7,64_ = 2.81, *P* < 0.00).

Interestingly, at pH 7.70, the organisms exposed to 16°C, 30°C, and 34°C, showed similar levels of hsp70 expression suggesting that stress can also occur at cooler temperatures. The concentration of hsp70 clearly decreased in the organisms from both pH treatments exposed to 36°C, indicating that these organisms cannot tolerate thermal stress due to high temperatures. In contrast, a state of homeostasis was found in the temperature range of 20 to 28°C as hsp70 expression was maintained at an average concentration of 0.028 ±0.002 ng mg^–1^.

**Fig 2:**
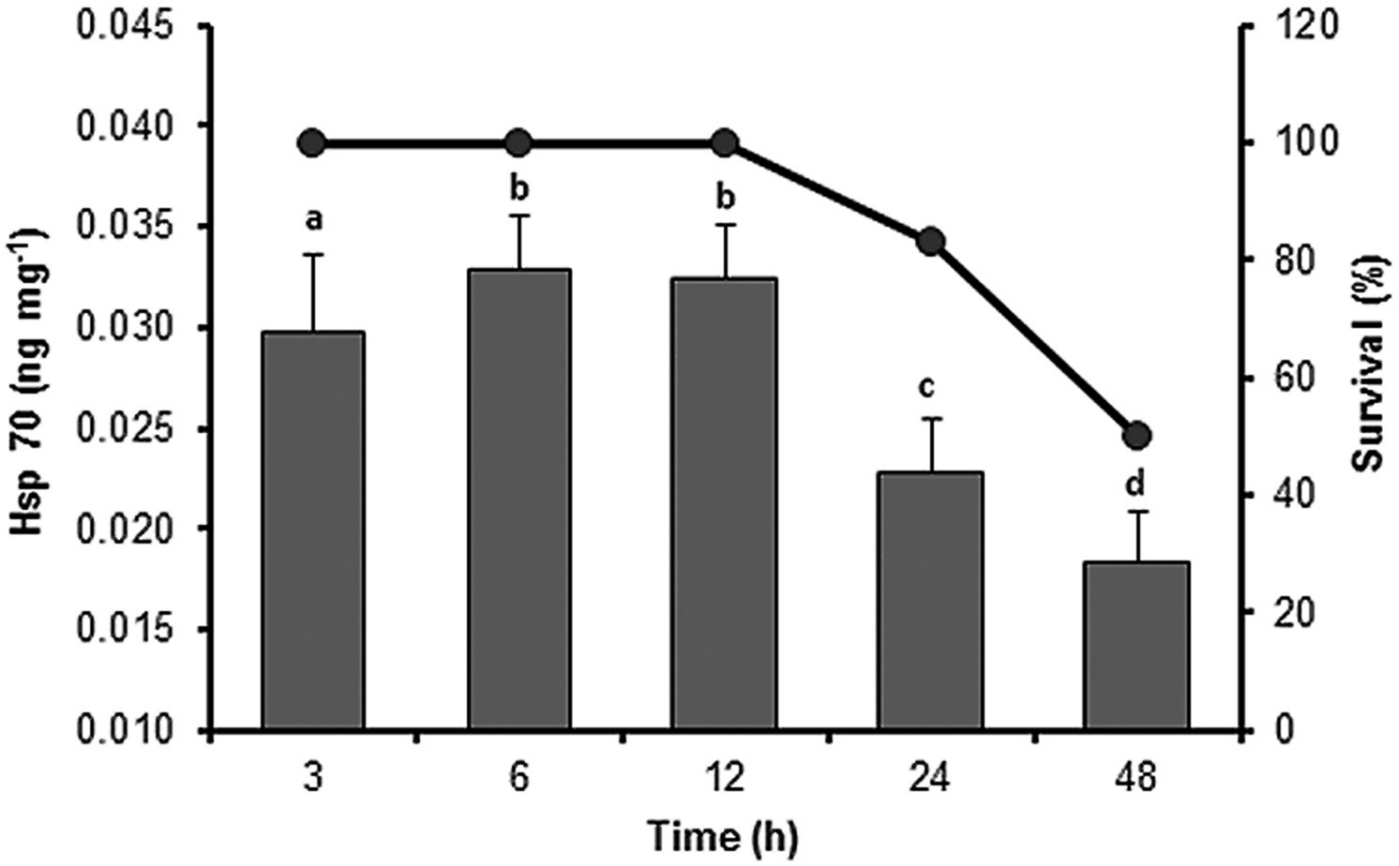
Expression of heat shock protein 70 (hsp70) from the sea cucumber *Isostichopus badionotus* in response to sublethal thermal tolerance. Line represents percentage survival; columns represent mean ±SE; n = 12 (factorial ANOVA; Tukey’s HSD, *P* < 0.05). Different letters represent significant differences between the exposure times.

The concentration of hsp70 increased upon 3−12 h of exposure to 34°C and reached a maximum of 0.0329 ±0.0026 ng mg^–1^ (*F*_4,50_ = 5.0, *P* = 0.0007; Fig 2). Subsequently, hsp70 expression decreased significantly after 24 h of exposure, coinciding with the beginning of mortality (17%) with the critical point being 48 h of exposure when mortality reached 50%.

### Differential responses of antioxidant enzymes to thermal stress and pH

**Fig 3:**
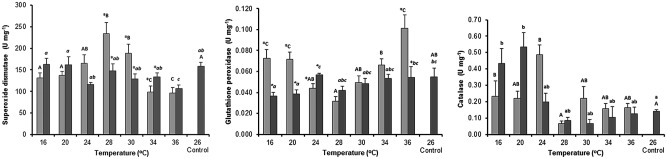
Interactive effect of pH and temperature on antioxidant activity in the sea cucumber *Isostichopus badionotus* exposed to cold–heat thermal stress for 6 h. Left = superoxide dismutase; Center = glutathione peroxidase; Right = catalase. ■ pH 8.17 ±0.02; ■ pH 7.70 ±0.08; control conditions: pH 8.17 ±0.02, 26 ±0.2°C. Columns represent mean ±SE; n = 5 with the exception of the control where n = 10 (factorial ANOVA; Tukey’s HSD, *P* < 0.05). The asterisks indicate significant differences between pH at the same temperature. Different letters (lower case for pH 8.17; capital letters for pH 7.70) represent significant differences between different temperature levels.

The interactive effects of temperature and pH on the activity of antioxidant enzymes (SOD, GPx, and CAT) of *I*. *badionotus* at 6 h of exposure are represented in Fig 3.

Alterations in pH significantly influenced the activity of SOD (*F*_7,64_ = 7.1, *P* < 0.0001) in the organisms exposed to 28, 30, or 34°C. SOD activity was higher at pH 7.70, particularly in the organisms exposed to 28°C (233.6 ±26.1 U mg^–1^) and 30°C (188.5 ±21.2 U mg^–1^), probably as part of a reaction to offset oxidative damage (Fig 3A). SOD activity fell below control values (158.2 ±8.80 U mg^–1^) upon exposure to 34 and 36°C at pH 7.70, and at 36°C in pH 8.17, suggesting reduced regulation of oxidative stress reactions. Mortality, however, only occurred in the organisms from both pH treatments exposed to 36°C. Organisms from both pH treatments exposed to the coldest temperatures (16 to 24°C) showed basal SOD activity (141.7 ±18.8 U mg^–1^) that was not different from control (158.2 ±8.80 U mg^–1^).

GPx activity was significantly influenced by pH (*F*_7,64_ = 8.0, *P* < 0.0001) in the organisms exposed to colder temperatures (16 to 24°C), and in the organisms exposed to 36°C (Fig 3B). GPx activity in the organisms exposed to 16 and 20°C was higher at pH 7.70 (mean 0.072 ±0.007 U mg^–1^) than at pH 8.17 and in the controls. Colder temperatures had an opposite effect at pH 8.17, as GPx activity (0.038 ±0.003 U mg^–1^) was significantly lower than control (0.057 ±0.008 U mg^–1^). The differential effects of pH were also evident at the extreme temperature of 36°C at which the organisms exposed to pH 7.70 showed the highest GPx activity (0.102 ±0.013 U mg^–1^). This interaction between lower pH and extreme temperatures appears to be the triggering factor for cellular oxidative stress. In organisms exposed to pH 8.17, GPx activity was not higher in the warmer temperatures (24 to 34°C = mean 0.052 ±0.005 U mg^–1^) and it was not different from control values (0.057 ±0.008 U mg^–1^).

CAT activity was affected by temperature (*F*_7,64_ = 3.73, *P* = 0.003; Fig 3C) but not by pH (*F*_1,64_ = 0.53, *P* = 0.54). In particular, at lower temperatures (16, 20, and 24°C) the activity of CAT was on average 2.5-fold higher than the control (0.140 ±0.012 U mg^–1^). In organisms exposed to temperatures greater than 26°C, CAT activity (0.116 ±0.011 U mg^–1^) was not significantly different from control.

### Sublethal thermal tolerance and antioxidant enzymes at different pH values

**Fig 4:**
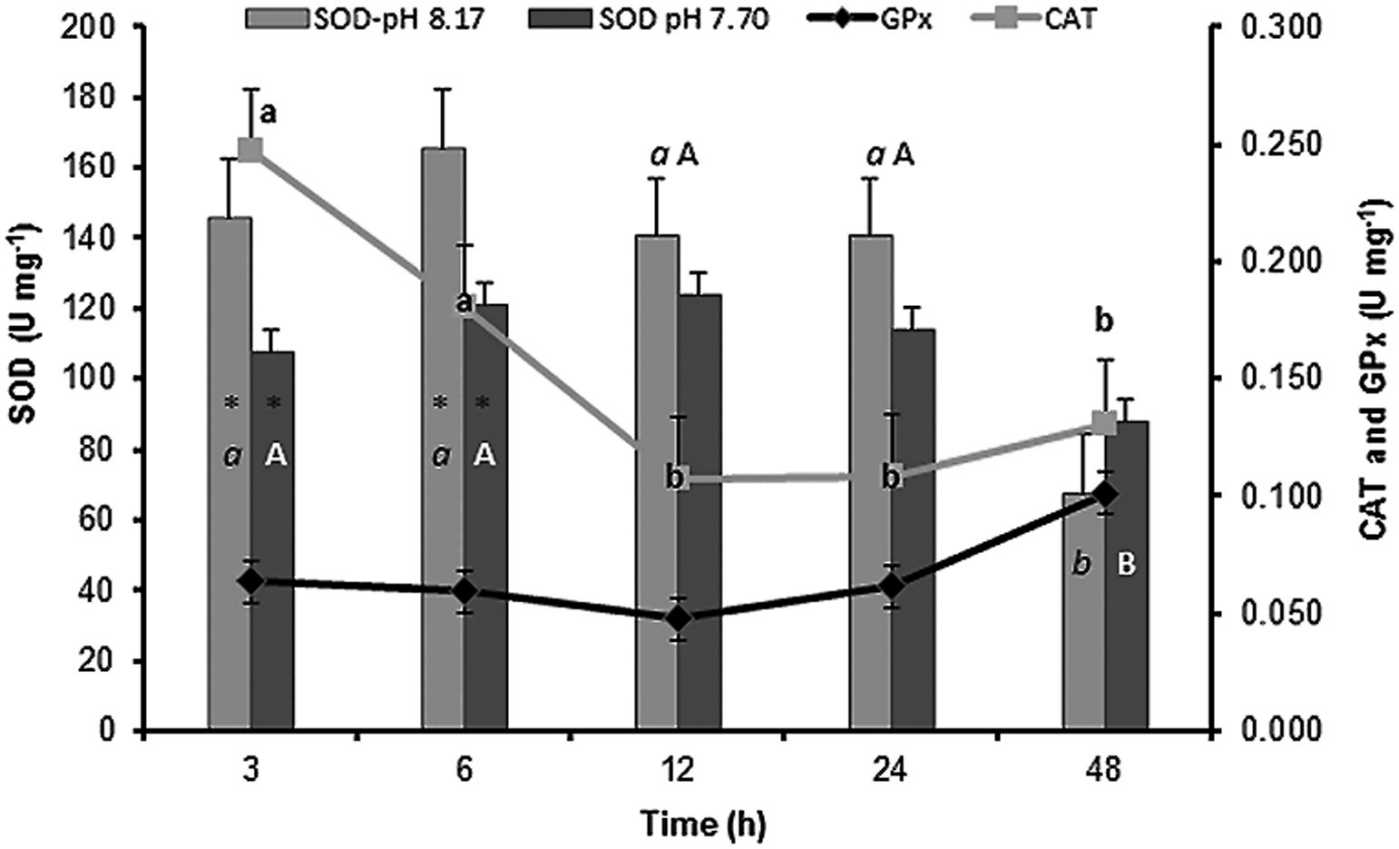
Sublethal thermal tolerance (34°C) from the sea cucumber *Isostichopus badionotus* exposed to different pH. Columns represent mean ±SE; n = 6 (factorial ANOVA; Tukey’s HSD, *P* < 0.05). ■ pH 8.17 ±0.02; ■ pH 7.70 ±0.08; control conditions: pH 8.17 ±0.02, 26 ±0.2°C. The asterisks indicate significant differences between pH values at the same temperature. Different letters (lower case for pH 8.17; capital letters for pH 7.70) represent significant differences between the exposure times at the same pH.

The interaction between exposure time and pH at the sublethal temperature (34°C) had no significant effect on the activity of GPx and CAT; therefore, data from these enzymes were analyzed as an average of temperature alone. GPx activity was upregulated at 48 h of exposure and reached a value of 0.101 U mg^–1^ (*F*_4,50_ = 6.50, *P* < 0.0001; Fig 4).

GPx activity was relatively stable between 3 and 24 h of exposure. The mean value for this period (0.058 ±0.009 U mg^–1^) was similar to the activity measured previously in the thermal limit experiment when organisms were exposed to 34°C for 6 h (0.060 U mg^–1^; Fig 3). This observation confirms that GPx activity at the sublethal temperature is affected only after 48 h of exposure.

Maximum CAT activity occurred at 3 h of exposure (0.247 ±0.030 U mg^–1^) and it was almost 2-fold greater than that registered at 6 h in the previous experiment (0.130 U mg^–1^; Fig 3). These data indicate that CAT activity is extremely sensitive to exposure times of 3−6 h. After 12 h of exposure, CAT activity significantly decreased to 0.131 ±0.028 U mg^-1^ (*F*_4, 50_ = 4.04, *P* < 0.004; Fig 4).

SOD activity was higher at pH 8.17 than at pH 7.70 and after 3 and 6 h of exposure (*F*_4,50_ = 4.29, *P* = 0.04; Fig 4). SOD activity decreased significantly after 48 h of exposure to 77.8 U mg^–1^. Thus, SOD and GPx activity showed opposing patterns with increasing exposure time.

### Effect of thermal stress on the basal activity of immune enzymes

**Fig 5:**
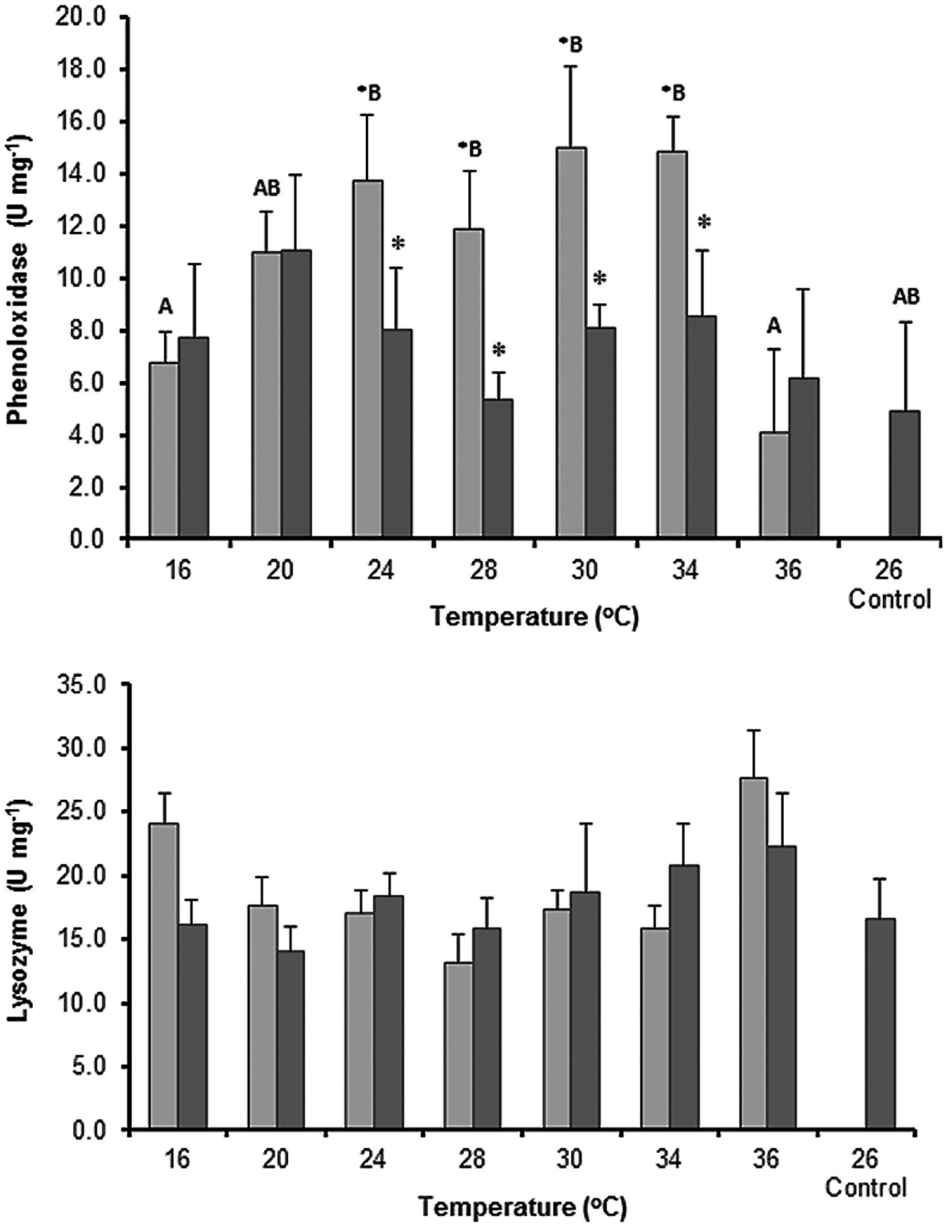
Effect of temperature on the basal activity of phenoloxidase and lysozyme from the sea cucumber *Isostichopus badionotus* exposed to two water pH values. ■ pH 8.17 ±0.02; ■ pH 7.70 ±0.08; control conditions: pH 8.17 ±0.02, 26 ±0.2°C. Columns represent mean ±SE; n = 5 with the exception of the control where n = 10 (factorial ANOVA; Tukey’s HSD, *P* < 0.05). Different letters (lower case for pH 8.17; capital letters for pH 7.70) represent significant differences between temperature levels.

PO activity was significantly influenced by pH (*F*_1,64_ = 3.1, *P* = 0.001) in the organisms exposed to temperatures between 24 and 34°C (Fig 5). Temperature had no effect on PO activity in the organisms exposed to pH 8.17 (*F*_7,64_ = 0.88, *P* = 0.53; Fig 5A). In contrast, at pH 7.70, the PO activity was higher at warmer temperatures (24, 28, 30, and 34°C) and increased 9-fold compared to the control. The PO activity decreased to 4.10 ±3.13 U mg^–1^ at 36°C, and pH had no influence on the PO activity at colder temperatures (16–20°C).

Neither pH nor temperature affected the basal activity of LSZ in the organisms (*F*_7,64_ = 460.7, *P* = 0.159). The mean LSZ activity at pH 7.70 was 18.9 ±2.26 U mg^–1^ and was 18.0 ±3.0 U mg^–1^ at pH 8.17. This enzyme showed minimum activity at 28°C and pH 7.70 (13.2 ±2.29 U mg^–1^) and maximum activity at 36°C (27.5 ±3.8 U mg^–1^) (Fig 5B).

## Discussion

The results of the present study clearly identify the upper lethal thermal limits of the tropical sea cucumber *I*. *badionotu*s. The organisms survived at higher temperatures of 30 and 34°C, but not at 36°C, implicating 34°C as the 12 h-sublethal temperature and 36°C as the 6 h-lethal temperature. As no mortality was seen at 16°C, the lower lethal limit could not be recorded but the high tolerance of *I*. *badionotus* to cold temperatures was confirmed. While changes in pH did not affect the biochemical parameters tested at lethal temperature, significant effects were seen at temperatures below 34°C. Most proteins, and therefore enzymes, are active only within a narrow intracellular pH (pH_i_) range, usually between pH 5 and 9 [25].

Hsp expression, which is considered as a bioindicator of thermal stress in sea cucumbers [26], significantly increased at 30–34°C, confirming that this temperature range induces thermal stress in *I*. *badionotus* (Fig 1). After 24 and 48 h of exposure at the sublethal temperature, the organisms in both pH treatments showed a significant decline in hsp70 and SOD activity, suggesting a disruption of functional homeostatic equilibrium (Figs 2 and 4). The decrease in hsp70 expression reported here is consistent with that reported for *A*. *japonicus* by Meng et al., who demonstrated that hsp70 expression declined in the organisms exposed to high temperatures [27]. The authors also associated this down-regulation of hsp70 with a breakdown in total protein synthesis and indicated that a decrease in protein synthesis determines the upper thermal limit of the sea cucumber *A*. *japonicus*. As the ability to withstand thermal stress for long time periods depends on complex mechanisms of stress tolerance, our data suggest that *I*. *badionotus* is extremely tolerant to cold temperatures and maintains homeostasis in the temperature range of 16–30°C.

An interesting finding of our study was the negative correlation among the antioxidant enzymes that modulate the effects of oxidative stress at various temperatures. This finding explains the ability of *I*. *badionotus* to withstand low temperatures for a long time despite its natural habitat being tropical. In particular, the functional relationship between the activity of SOD and GPx in regulating a cellular redox homeostasis is a critical regulator of thermal stress [14]. In the present study, the activity of GPx was maximal at the extreme cold and warm temperature tested (16, 20 and 36°C), contrary to that seen for SOD at identical pH levels. A similar situation occurred with CAT activity, and although its activity was not influenced by the effect of pH, its concentration was significantly higher at colder temperatures (16 and 24°C). The dynamics of antioxidant enzymes in invertebrates, particularly in sea cucumbers, have been poorly studied. Nonetheless, reports in fish demonstrate that increasing temperature from 25 to 37°C for 1 to 4 h raised SOD activity and reduced the levels of GPx in the catfish *Heteropneustes fossilis* [28]. While the upregulation of SOD with increasing temperature is probably associated with a role for scavenging O_2_^−^, the gradual depletion of GPx is most likely associated with an increased risk of oxidative stress as high SOD would increase the peroxidative burden and increase lipid peroxidation. In the case of CAT activity, an inverse correlation with SOD has been reported in the clam *Macoma balthica* upon exposure to trace metals [29]. As the increased activity of antioxidant enzymes suggests an active production of ROS as a consequence of the activation of the NADH oxidase system and an increase in oxygen consumption [30], the change in SOD activity due to the interactive effect of thermal stress and pH 7.70 could be associated with a high metabolic rate. At extreme temperatures, however, the increase in the activity of GPx argues for a protective role of this enzyme against thermal shock. We believe that the inverse correlation between antioxidant enzymes reflects the need to preserve redox balance in stressful situations in *I*. *badionotus*. The enzymatic components of the antioxidant system possess different target sites and functions within the cells. SOD catalyzes the dismutation of O_2_^−^ into either oxygen O_2_^−^ or H_2_O_2_; H_2_O_2_ is then detoxified by two different systems, either the GPx system, which is the first line of defense against H_2_O_2_ or CAT, which is the secondary system. Increased GPx and CAT activity indicates the presence of high H_2_O_2_ concentrations within cells, and this could act as a regulator of SOD activity at specific temperatures.

Overall, the mean activity of the three antioxidant enzymes was higher at pH 7.70 than at pH 8.17. SOD increased significantly but only between 28 to 30°C, probably as part of a reaction to offset the oxidative damage, while GPx activity increased at both colder temperatures of 16 to 24°C and at a higher temperature of 36°C. The impact of decreasing pH on the aerobic scope and the metabolic rate has been assessed in several fish species, and the prevailing hypothesis is that elevated *p*CO_2_ increases the metabolic rate, causes a shift in energy budget, and reduces aerobic scope [31]. In general, vertebrates are excellent osmotic and acid-base regulators and are, therefore, better able to cope with pH changes compared to invertebrates, which have a lower regulation capacity [32]. There are only a few reports on the effects of pH on antioxidant enzymes in invertebrates, and such data are scarce in the sea cucumber. Interestingly, in the oyster *Crassostrea virginica*, exposure to pH 7.5 induced oxidative stress [33], and the authors suggested that low pH caused oxidative stress by increasing the production of ROS, both indirectly by lowering internal pH and probably enhancing the Fenton reaction, and directly by CO_2_ interacting with other ROS to form more free radicals. Hu et al. studied the combined effect of acidification and temperature on the antioxidant responses of *Mytilus coruscus* and concluded that SOD, GPx, and CAT were significantly increased when water pH decreased from 8.10 to 7.30 [34]. Our results point towards a similar effect in the sea cucumbers exposed to pH 7.70, with an increase in metabolic rate and antioxidant enzyme activity as the first line of defense against the harmful effects of ROS. Our data also show that, at 36°C, the organisms lost their ability to maintain the intracellular redox homeostasis, although GPx activity increased and SOD activity decreased to 101.2 U mg^–1^. Further, at the sublethal temperature, the activity of SOD seems to be crucial for controlling oxidative stress, at least in the first 24 h of exposure, and GPx is upregulated belatedly, possibly as a consequence of SOD depletion.

The epidermal layer of the sea cucumber *I*. *badionotus* was found to be an important defense barrier that was activated under the effect of environmental parameters, particularly, water pH. Mucosal immunity was evidenced through activation of the PO system, which increased from basal levels at pH 7.70 and at warm temperatures (28, 30, and 34°C). This result is in accordance with data reported by Wang et al., who demonstrated that heat stress significantly increases the cellular response and phagocytic activity of CL in the cells from *A*. *japonicus* over a short period of time (0.5–3 h) [7]. However, in contrast to our findings, Wang et al. also reported an increment in LSZ activity in the temperature range from 0 to 16°C and a decrease at 32°C after 6 to 12 h of exposure, suggesting non-optimal conditions [7]. We show that LSZ activity increased with an increase in temperature, particularly at pH 8.17, but that its concentration was not different from control (26°C = 16.5 ±3.2 U mg^–1^). The range of LSZ activity determined in the present study (13.2 to 27.5 U mg^–1^) agrees with our previous results in which *I*. *badionotus* exposed to pH 8.13 and 25 ± 1°C had an LSZ level of 15.8 ±4.7 U mg^–1^ in the CL [5]. Thus, contextually, no differences in LSZ activity were observed in the mucus and CL of unstimulated sea cucumbers, but the stimulatory effect of pH was an unexpected result. Nevertheless, we agree that LSZ activation in *I*. *badionotus* requires multiple immunostimulants and not just changes in ambient pH [35]. Finally, it is interesting to note that at pH 8.17, the basal activity of PO ranged between 5.31 and 11.0 U mg^–1^, which is higher than that reported previously by Gullian-Klanian in the CL (2.86 U mg^–1^ protein) of wild *I*. *badionotus* exposed to the same pH [14]. This finding suggests that the epidermal PO system probably plays an important role in the immune response of *I*. *badionotus*, which is an activated barrier to bacterial invasion when organisms are exposed to the stressful environmental factors.

In summary, *I*. *badionotus* is a eurythermal organism capable of tolerating a wide range of temperatures from 16 to 34°C at pH 7.70. Environmental conditions for optimal physiological performance were found to be pH 8.17 and a temperature range of 24–28°C. The interaction between the two the stressors (temperature and pH) activated defense systems such as antioxidant enzymes and mucosal PO, indicating that *I*. *badionotus* is well-adapted to tolerate extreme environmental stressors, at least for a short period of time. An alteration in ambient pH was a stressful condition that stimulated the activation of antioxidant enzymes at various critical thermal limits as an effort to protect intracellular redox homeostasis. *I*. *badionotus* showed signs of thermal stress by synthesizing hsp70 at extreme cold (16°C) and warm (34°C) thermal limits, temperatures at which the effect of pH was not significant.

## Supporting information

S1 Table Thermal limits data set. (XLSX)

S2 Table Thermal tolerance data set. (XLSX)
